# Two-sample Mendelian randomization study does not reveal a significant relationship between cytomegalovirus (CMV) infection and autism spectrum disorder

**DOI:** 10.1186/s12888-023-05035-w

**Published:** 2023-08-02

**Authors:** Mengna Zhang, Ying Ming, Yunling Du, Ziyuan Xin

**Affiliations:** Molecular Diagnosis Center, The Affiliated Hospital of Chengde Medical University, Chengde, HeBei Province 067000 China

**Keywords:** Autism spectrum disorders (ASD), Cytomegalovirus (CMV) infection, Congenital CMV infection, 2-sample Mendelian randomization

## Abstract

**Background:**

Autism spectrum disorder (ASD) is a neurodevelopmental disorder affecting ~ 2% of children worldwide and is characterized by repetitive, stereotypical behaviours and impaired expressive communication. Cytomegalovirus (CMV) is considered a risk factor for ASD; however, published studies are usually limited by covering too few events and have different conclusions, indicating that the relationship between CMV infection and ASD remains elusive.

**Methods:**

To investigate the association between CMV infection and ASD, we conducted this 2-sample Mendelian randomization (MR) study using genome-wide association studies (GWAS) summary data from FinnGen and the IEU Open GWAS project.

**Results:**

Our results showed no significant relationship between all 3 CMV infections (unspecified cytomegaloviral diseases, anti-CMV IgG levels, and maternal CMV) and ASD.

**Conclusions:**

Our results indicate that CMV infection does not significantly increase ASD risk. These results show that the relationship between CMV infection and ASD remains elusive and needs to be further clarified.

**Supplementary Information:**

The online version contains supplementary material available at 10.1186/s12888-023-05035-w.

## Background

Autism spectrum disorder (ASD) is a neurodevelopmental disorder characterized by repetitive, stereotypical behaviours and impaired expressive communication [1]. The prevalence of ASD is estimated to be ~ 2% in developed countries [2] and relatively lower in lower-income areas [3]. The aetiology of ASD remains elusive; however, many risk factors for ASD have been discovered, including genetic and environmental factors [4].

Cytomegalovirus (CMV) is considered a risk factor for ASD since CMV-infected infants can manifest multiple central nervous system (CNS) disorders, such as microcephaly, periventricular calcifications, and sensorineural hearing loss [5]. As a widely known virus affecting the CNS in paediatric patients, CMV can be transmitted from pregnant mothers to newborns, which is defended as congenital CMV infection. Postnatal infections are usually transmitted by exposure to human milk, blood products, or transplanted organs. Most congenital infections and human milk-associated CMV infections are asymptomatic [5, 6].

CMV infection has been　suggested as a risk factor for ASD for many decades,　but systematic studies on the correlation between CMV infections and ASD are still limited. There are some case series that reported that congenital CMV increased the risk of ASD; however, the sample sizes of these studies are very small (n < 10) [7, 8]. A meta-analysis showed a high prevalence of congenital CMV infection in ASD cases, but this result was still limited by having too few events (0–2) in all included studies [9]. The titer and seropositivity rates of antibodies to CMV are similar between children with ASD and healthy controls [10]. Moreover, a 12-year retrospective analysis revealed that cognitive and motor development were similar in extremely premature infants with or without postnatal CMV [11]. These studies showed that the relationship between CMV infection and ASD remains unclear and needs to be further clarified.

Mendelian randomization (MR) is a method that uses variants that are robustly associated with exposure factors to generate more reliable evidence regarding which interventions should influence specific outcomes [12]. Evidence derived from MR has a lower strength than randomized controlled clinical trials (RCTs) but is stronger than observational studies, such as case‒control studies [13]. On the other hand, MR studies usually include a much larger sample size than clinical trials, especially when there is a low clinical prevalence.

In this study, we used 2-sample MR to investigate the association between CMV infection and ASD. We used genetic variants associated with unspecified cytomegaloviral diseases, anti-CMV IgG levels, and maternal CMV as instrumental variables to evaluate CMV infection as a risk factor for ASD.

## Methods

**Fig. 1:**
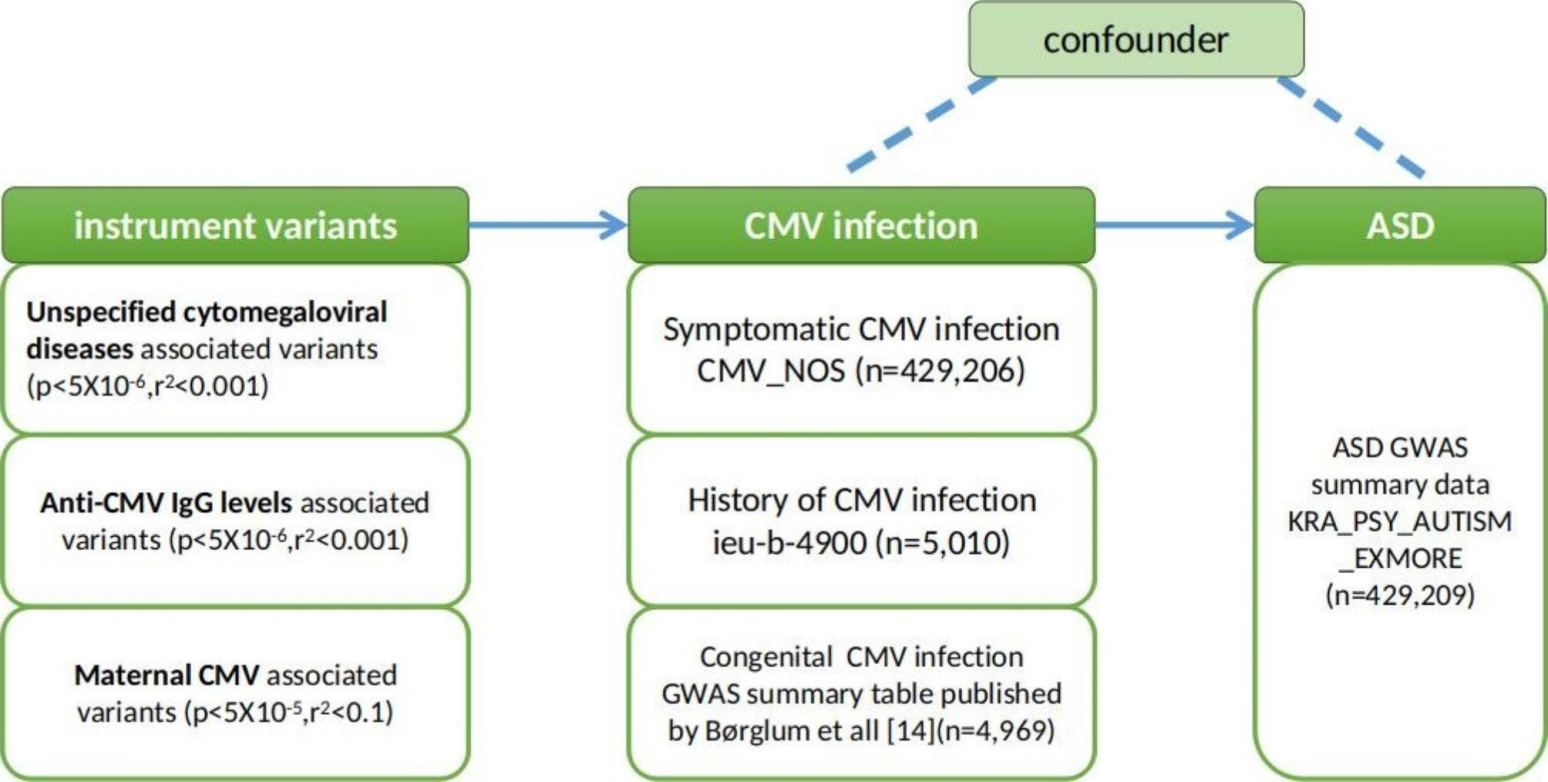
The workflows of instrument variant selection and MR analysis

The workflows of instrument variant selection and MR analysis are shown in Fig. 1.

### Data sources

We conducted this 2-sample MR study using genome-wide association studies (GWAS) summary data from FinnGen and the IEU Open GWAS project. FinnGen (https://www.finngen.fi) is a large public‒private partnership aiming to collect and analyse genome and health data from 500,000 Finnish biobank participants. The GWAS data are combined with phenotype data produced from several national health registries. The IEU Open GWAS project (https://gwas.mrcieu.ac.uk/) is an open-source data infrastructure that contains 126 billion genetic associations from 14,582 complete GWAS datasets representing a range of different human phenotypes and disease outcomes across different populations [14].

1. Instrumental variables of unspecified cytomegaloviral diseases were extracted from FinnGen GWAS summary data CMV_NOS (n = 429,206) (https://r8.finngen.fi/pheno/CMV_NOS).
2. Instrumental variables of anti-CMV IgG levels were extracted from the IEU Open GWAS project. The GWAS summary dataset ieu-b-4900 (n = 5,010) (https://gwas.mrcieu.ac.uk/datasets/ieu-b-4900/) was used to investigate the association between CMV infection and ASD. The population of this study was European.
3. Instrumental variables of maternal CMV were extracted from the GWAS summary table published by Børglum et al [15](n = 4,969, only variants with P < 5 × 10^− 4^ are listed). The population of this study was German-Dutch.

GWAS summary data KRA_PSY_AUTISM_EXMORE (n = 429,209) from FinnGen were used as the outcome dataset in MR (https://r8.finngen.fi/pheno/KRA_PSY_AUTISM_EXMORE). The linkage disequilibrium (LD) reference was obtained from http://fileserve.mrcieu.ac.uk/ld/1kg.v3.tgz.

### Statistical analysis

Two-sample MR [16] uses genetic variants extracted from GWAS summary data as instruments to assess the causal relationship between exposure and outcome. We chose instrument variants (IVs) by following these steps: (1) Select the genetic variant with the lowest p value. (2) Remove all other genetic variants from consideration that are correlated with the chosen variant. (3) Select the genetic variant with the lowest p value from the remaining variants and again remove variants correlated with the chosen variant [17].

In these 3 MR analyses, IVs in (1) and (2) were selected by P < 5 × 10^− 6^ and r2 < 0.001. In study (3), IVs were selected by P < 5 × 10^− 5^, and r2 < 0.1 for no variants in this study met our former criteria. To evaluate weak instruments used in MR analysis, the instrument strength (F-statistic) for every single variant was calculated as follows:

F = (beta/se(beta))^2^ [18].

All 2-sample MR analyses were conducted by R v4.2.2 and R package TwoSampleMR v0.5.6 (https://mrcieu.github.io/TwoSampleMR). Variant clumps were generated by the R package ieugwasr v0.1.5 (https://mrcieu.github.io/ieugwasr). Variants LD with other variants, absence from LD reference, or palindromic variants were trimmed before MR analysis. MR‒Egger regression, weighted median, weighted mode, simple mode, and inverse variance weighted (IVW) were used as MR methods.

### A positive control MR

To further assess whether our IVs are informative or not sufficiently informative to reveal a causal association, we conducted a positive control MR analysis between CMV and known CMV-associated symptoms,hydrocephalus [19] (other and unspecified hydrocephalus, finn-b-G6_HCOTHUNS,https://gwas.mrcieu.ac.uk/datasets/finn-b-G6_HCOTHUNS/). This positive control is described in detail in Additional file 1.

## Results

### Genetic instruments

**Fig. 2:**
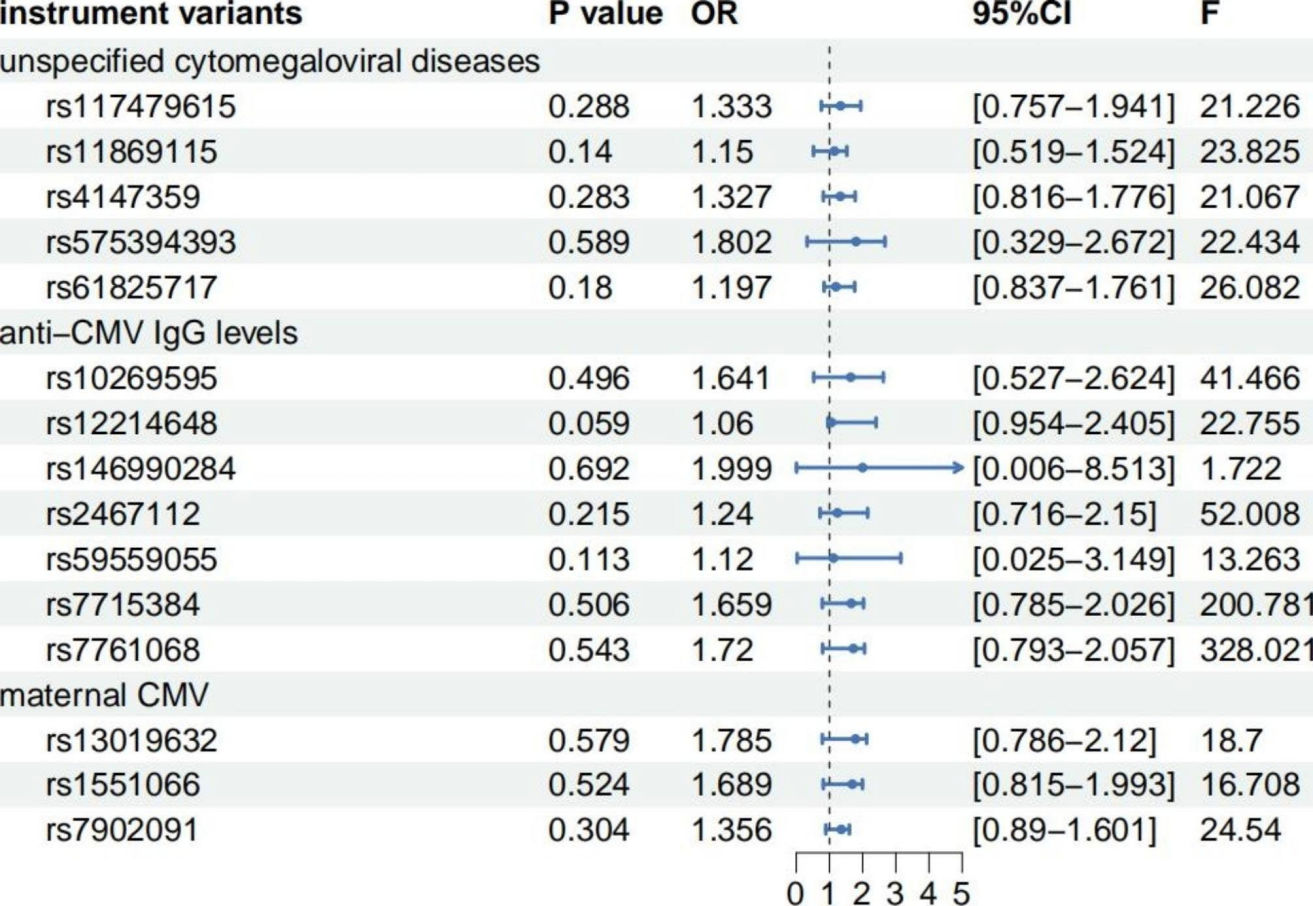
Variants used as IVs in this study

Following the parameters described above, 5 variants were used as IVs for unspecified cytomegaloviral diseases, 7 variants for anti-CMV IgG levels, and 3 variants for maternal CMV (Fig. 2). The LD matrix between SNPs and the statistical values of SNPs in the exposure and outcome are available in Additional files 2 and 3.

### Causal estimation of CMV infection on ASD

**Fig. 3:**
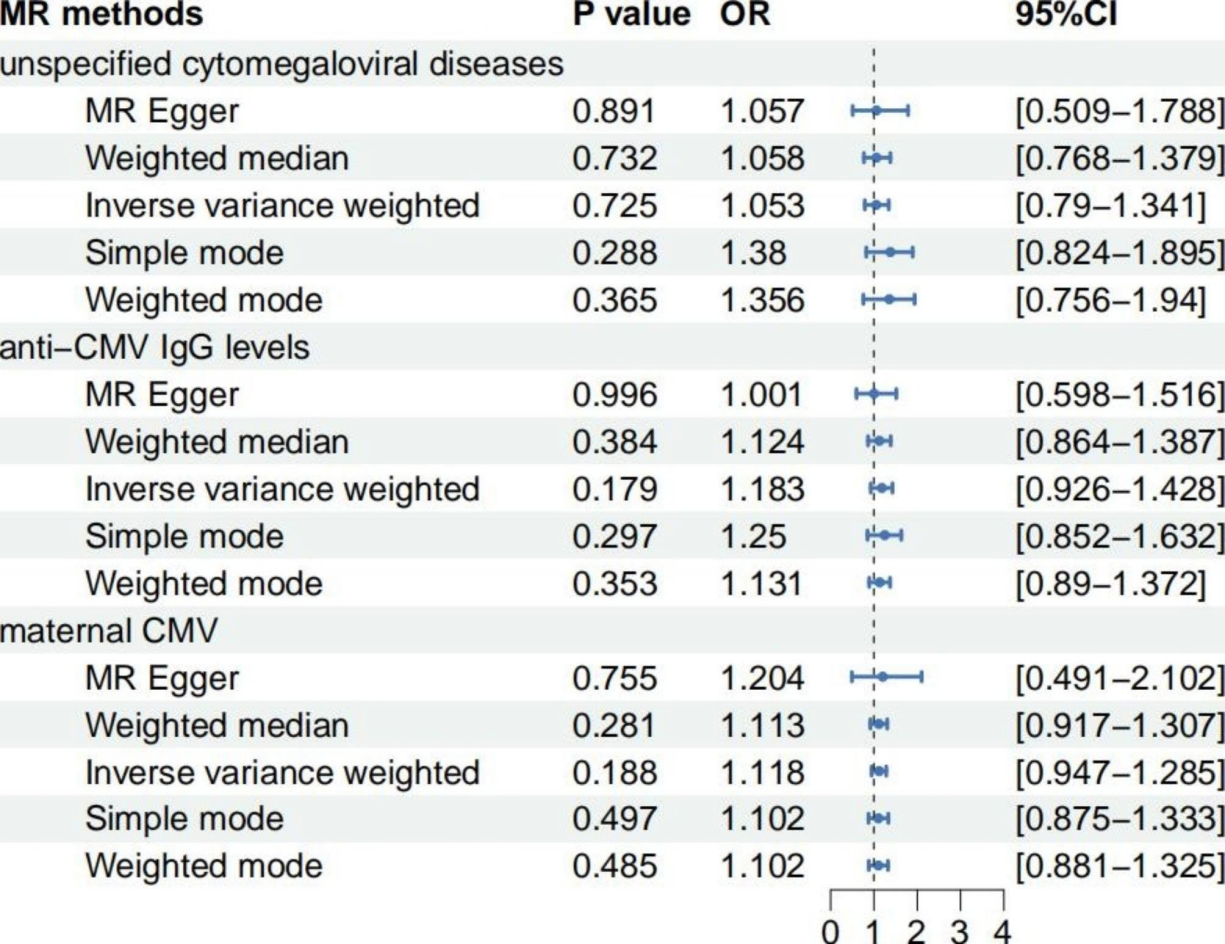
MR analysis of CMV infections and ASD.

Based on GWAS summary datasets from FinnGen and the IEU Open GWAS project, MR analysis was conducted to assess the relationship between 3 kinds of CMV infections and ASD. All static methods showed no significant causal relationship between CMV infections and ASD (Fig. 3). Only 1 IV might have relationship with ASD (P = 0.059, rs12214648, Fig. 2).

### Pleiotropy, heterogeneity, and instrument strength for MR estimates

MR‒Egger regression and IVW showed no evidence for heterogeneity and horizontal pleiotropy (Table 1). Pleiotropy refers to a genetic variant being associated with multiple risk factors for the outcome. This could be evaluated by the interception of Egger regression (Egger intercept column of Table 1). All intercepts were near 0, indicating that the IVs were not additionally associated with another risk factor for the outcome.

**Table 1:** Heterogeneity and horizontal pleiotropy of 3 MR studies

exposure	method	Qpval	Egger intercept	pval
**unspecified cytomegaloviral diseases**	MR Egger	0.096	-0.002	0.991
	IVW	0.174		
**anti-CMV IgG level**	MR Egger	0.278	0.056	0.500
	IVW	0.324		
**maternalCMV**	MR Egger	0.929	-0.044	0.896
	IVW	0.983		

In all 3 CMV infection-associated GWAS datasets, none of the variants met statistical significance P < 5 × 10^− 8^. In study (3), we set the criteria to P < 5 × 10^− 5^ and r2 < 0.1 to avoid single IV in this MR analysis. The F-statistic for each IV is listed in Fig. 2. All variants but 1 (rs146990284) were not too weak instruments (F > 10).

We described a positive control MR analysis in Additional file 1 to show that our IVs are informative to indicate a causal relationship between CMV infection and hydrocephalus.

## Discussion

ASD affects people worldwide and brings both health and economic burdens. As a widely known virus affecting the CNS in paediatric patients, CMV has long been suggested as a risk factor for ASD, yet no definite evidence has been found. To investigate whether CMV infection has a causal relationship with ASD, we conducted 3 MR analyses between ASD and unspecified cytomegaloviral diseases, anti-CMV IgG levels, and maternal CMV using GWAS summary data from the FinnGen and IEU Open GWAS projects.

A total of 15 variants were used as IVs in these 2-sample MR studies. Limited by low clinical prevalence, no variants significantly associated with CMV infection (default cut-off used in MR was p < 5 × 10^− 8^) were identified in these GWAS datasets. This normally used threshold of 5 × 10^− 8^ would predict an F statistic of approximately 30, and a higher F statistic means lower bias. However, all IVs but 1 (rs146990284) had an F over 10, indicating that they were not too weak instruments.

MR Egger regression, IVW, weighted median, weighted mode and simple mode were used in MR analysis. The results showed no causal relationship between CMV infection and ASD, indicating that CMV IgG positivity could not be considered a risk factor for ASD. Mendelian randomization studies could derive evidence with stronger strength than observational studies with a much larger sample size [13],thus partly covering the shortage of previously published studies with too few events (0–2) included.

While qualitative studies were limited by event number, quantitative studies were published. Slawinski et al. reported that maternal CMV seropositivity was related to autism symptoms (n = 82 and 68 for CMV-negative and CMV-positive groups,respectively) [20]. They used the Social Responsiveness Scale, Second Edition (SRS-2) as the ASD evaluation method, and in the maternal CMV sero-positive group, the mean score was 54.8, which was higher than that of the negative group (51.1). However, the population mean of the SRS-2 was expected to be 50, with a standard deviation of 10, and in ASD diagnosis, it was used as a categorical outcome (< 60 indicated normal and 60–65 indicated mild deficits in social interactions). This study indicates that CMV infection might contribute to a small part of ASD risk factors, which is not enough for a categorical outcome.

The main limitation of this study is the small number of IVs, and most IVs are not strong instruments, which is a consequence of the low clinical prevalence of CMV infection. A large number of IVs with strong evidence strength is not always available, and a small number of IVs with strong and specific biological links could also provide evidence. To further assess whether our IVs are informative or not sufficiently informative, we conducted a positive-control MR analysis between CMV and hydrocephalus [19] (Additional file 1). A significant causal relationship was found between unspecified cytomegaloviral diseases (5 IVs) and hydrocephalus. All 5 IVs from unspecified cytomegaloviral disease GWAS summary data had an F-statistic of approximately 20. However, the maternal CMV dataset with fewer IVs (3 IVs) did not have a positive result in the positive control. The negative results might be a consequence of a low proportion of symptomatic congenital CMV. Approximately 80–90% of CMV infections are asymptomatic [5, 6]. If only severe infections contribute to the outcome, then the number of events would be very low; consequently, a very large sample size is needed to assess the risk. These factors showed another limitation in this study. Asymptomatic CMV infection and infection with severe symptoms were not distinguished in the exposure datasets. Study 1 with an unspecified cytomegaloviral disease (symptomatic CMV infection) dataset could partly cover this shortage. The combination of all 3 studies could increase the sensitivity of finding causal relationships between CMV infection and other clinical symptoms.

Our results provide evidence that CMV infection does not directly increase ASD risk by 2-sample MR, and this evidence is stronger than observational studies. These results, together with published literature, show the complexity of ASD aetiology. Researchers should be more cautious when analysing the association between CMV infection and ASD. To further investigate the contribution of CMV infection to ASD aetiology, population-based studies with large sample sizes are needed, as is a better-designed attribution analysis.

## Electronic supplementary material

Below is the link to the electronic supplementary material.

Supplementary Material 1

Supplementary Material 2

Supplementary Material 3

## Data Availability

All data used in this study are obtained from open access databases or published manuscript. FinnGen: https://r8.finngen.fi/pheno/CMV_NOS. https://r8.finngen.fi/pheno/KRA_PSY_AUTISM_EXMORE. IEU Open GWAS project: https://gwas.mrcieu.ac.uk/datasets/ieu-b-4900. https://gwas.mrcieu.ac.uk/datasets/finn-b-G6_HCOTHUNS.

## List of Abbreviations

ASD: Autism spectrum disorders
CMV: Cytomegalovirus
GWAS: Genome-wide association studies
IVs: Instrument variants
IVW: Inverse variance weighted
LD: Linkage disequilibrium
MR: Mendelian randomization
RCTs: Randomized controlled clinical trials
