# Supplementary material for: Two-sample Mendelian randomization study does not reveal a significant relationship between cytomegalovirus (CMV) infection and autism spectrum disorder
Source: BMC Psychiatry. 2023 Aug 2;23:559. doi: 10.1186/s12888-023-05035-w (PMC10394766; doi:10.1186/s12888-023-05035-w)
Supplement: Supplementary file 1 — Supplementary Material 1 [file 12888_2023_5035_MOESM1_ESM.doc]

**Additional file 1 A positive control.** MR analysis between CMV and hydrocephalus, a known CMV associated symptoms, using identical exposure datasets and IVs to the main body.

In this additional file we describe a positive control 2-sample Mendelian randomization (MR) study using instrument variants(IVs) chosen in the main body of the manuscript.

**Background**

In this positive control, we chose hydrocephalus as the outcome. Postinfectious hydrocephalus is considered the most common cause of pediatric hydrocephalus [1].

Population-based study showed cytomegalovirus (CMV) infection was significantly higher in postinfectious hydrocephalus than non-postinfectious hydrocephalus[2].

**Methods**

We use the same exposure datasets and IVs described in Methods of the main body.

The outcome dataset on hydrocephalus is obtained from IEU Open GWAS project (https://gwas.mrcieu.ac.uk/)[3]. This dataset about hydrocephalus (Other and unspecified hydrocephalus, finn-b-G6_HCOTHUNS,  <https://gwas.mrcieu.ac.uk/datasets/finn-b-G6_HCOTHUNS/>) is a European population-based GWAS summary with 173 cases and 218,043 controls.

The statistical pipeline of 2-sample MR is also described in Methods of the main body.

MR Egger regression, weighted median, weighted mode, simple mode, and inverse variance weighted (IVW) were used as MR methods. If at least one method found a potential relationship, we would further analyze the data by MR-Pleiotropy RESidual Sum and Outlier (MR-PRESSO)[4]. MR-PRESSO distortion test was used for testing significant distortion in the causal estimates before and after outlier removal.

**Results**

MR analysis was conducted to access the relationship between 3 kinds of CMV infections and hydrocephalus. The IVs used in these studies were all identical to the IVs used in the main body of the manuscript. A causal relationship was indicated between unspecified cytomegaloviral diseases and hydrocephalus (P=0.066 and 0.051, statics by weighted mode and IVW). MR-PRESSO showed a significant causal relationship between unspecified cytomegaloviral diseases and hydrocephalus (P=0.029). No static methods showed a significant causal relationship between anti-CMV IgG levels or maternal CMV and hydrocephalus.

No evidence for heterogeneity and horizontal pleiotropy was found in this study. All intercepts were near 0, indicating the IVs were not additionally associated with another risk factor for the outcome.


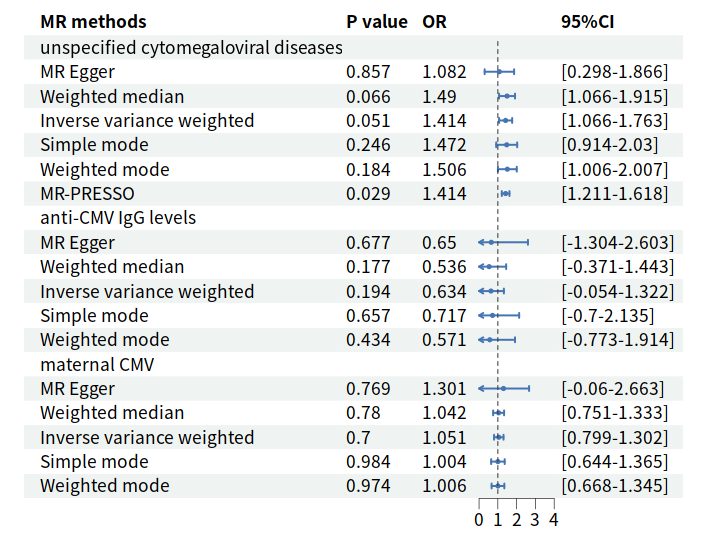


**Figure 1 MR analysis of CMV infections on hydrocephalus.**

**Discussion**

In this positive control, we described a significant causal relationship between unspecified cytomegaloviral diseases and hydrocephalus found by 2-sample MR, as MR is a method for causal inference using genetic variants. We selected 5 IVs from unspecified cytomegaloviral diseases and all of them did not have confounding factors associated with outcome (P>0.05 by using MR-Egger regression and IVW accessing horizontal pleiotropy). Weighted mode and IVW indicated a potential relationship between CMV infection and hydrocephalus (P=0.066 and 0.051, respectively), while MR‐Egger regression showed a negative result. In practice, the IVW estimate is likely to yield far more precise estimates than MR‐Egger regression[5]. MR-PRESSO showed a significant causal relationship between unspecified cytomegaloviral diseases and hydrocephalus (P=0.029). These results showed our IVs were informative for indicating a causal relationship.

The maternal CMV infection dataset had 3 IVs, leading to a big concern of less informative to reveal the relationship. The negative results might be a consequence of a low proportion of symptomatic congenital CMV. About 80-90% of CMV infections were asymptomatic[6,7]. If only severe infections contribute to the outcome, then the number of events would be very low, consequently a very large sample size is needed to access the risk. Study 1 with unspecified cytomegaloviral diseases (sympotic CMV infection) data set could partly cover this shortage.

Though the most reported symptoms of congenital CMV infection are sensorineural hearing loss[8], a population-based meta-analysis showed congenital CMV infection did not significantly increase the risk of sensorineural hearing loss[9]. So we did not choose sensorineural hearing loss as the outcome in this positive control study.

**Reference**

[1] Warf BC. Hydrocephalus in Uganda: the predominance of infectious origin and primary management with endoscopic third ventriculostomy. J Neurosurg. 2005;102(1 Suppl):1-15. doi:10.3171/ped.2005.102.1.0001

[2] Hehnly C, Ssentongo P, Bebell LM, et al. Cytomegalovirus infections in infants in Uganda: Newborn-mother pairs, neonates with sepsis, and infants with hydrocephalus. Int J Infect Dis. 2022;118:24-33. doi:10.1016/j.ijid.2022.02.005

**[3]** Elsworth B, Lyon M, Alexander T, Liu Y, Matthews P, Hallett J, et al. The MRC IEU OpenGWAS data infrastructure.bioRxiv 2020.08.10.244293v1. doi: 10.1101/2020.08.10.244293

[4] Verbanck M, Chen CY, Neale B, Do R. Detection of widespread horizontal pleiotropy in causal relationships inferred from Mendelian randomization between complex traits and diseases. Nat Genet. 2018 May;50(5):693-698. doi: 10.1038/s41588-018-0099-7.

[5] Bowden J, Holmes MV. Meta-analysis and Mendelian randomization: A review. Res Synth Methods. 2019 Dec;10(4):486-496. doi: 10.1002/jrsm.1346.

[6] Plosa EJ, Esbenshade JC, Fuller MP, Weitkamp JH. Cytomegalovirus infection. Pediatr Rev. 2012 Apr;33(4):156-63; quiz 163. doi: 10.1542/pir.33-4-156.

[7] Bryant P, Morley C, Garland S, Curtis N. Cytomegalovirus transmission from breast milk in premature babies: does it matter? Arch Dis Child Fetal Neonatal Ed. 2002 Sep;87(2):F75-7. doi: 10.1136/fn.87.2.f75.

[8] Goderis J, De Leenheer E, Smets K, Van Hoecke H, Keymeulen A, Dhooge I. Hearing loss and congenital CMV infection: a systematic review. Pediatrics. 2014 Nov;134(5):972-82. doi: 10.1542/peds.2014-1173.

[9] Maltezou PG, Kourlaba G, Kourkouni Ε, Luck S, Blázquez-Gamero D, Ville Y, et al. Maternal type of CMV infection and sequelae in infants with congenital CMV: Systematic review and meta-analysis. J Clin Virol. 2020 Aug;129:104518. doi: 10.1016/j.jcv.2020.104518.
